# Supplementary material for: scSemiProfiler: Advancing large-scale single-cell studies through semi-profiling with deep generative models and active learning
Source: Nat Commun. 2024 Jul 16;15:5989. doi: 10.1038/s41467-024-50150-1 (PMC11252419; doi:10.1038/s41467-024-50150-1)
Supplement: Supplementary file 2 — Reporting Summary [file 41467_2024_50150_MOESM2_ESM.pdf]

nature portfolio

Corresponding author(s): Jun Ding

Last updated by author(s): Jun 9, 2024

Reporting Summary

Nature Portfolio wishes to improve the reproducibility of the work that we publish. This form provides structure for consistency and transparency in reporting. For further information on Nature Portfolio policies, see our Editorial Policies and the Editorial Policy Checklist.

Statistics

For all statistical analyses, confirm that the following items are present in the figure legend, table legend, main text, or Methods section.

n/a	Confirmed	
☐	☒	The exact sample size (*n*) for each experimental group/condition, given as a discrete number and unit of measurement
☐	☒	A statement on whether measurements were taken from distinct samples or whether the same sample was measured repeatedly
☐	☒	The statistical test(s) used AND whether they are one- or two-sided<br>*Only common tests should be described solely by name; describe more complex techniques in the Methods section.*
☒	☐	A description of all covariates tested
☐	☒	A description of any assumptions or corrections, such as tests of normality and adjustment for multiple comparisons
☐	☒	A full description of the statistical parameters including central tendency (e.g. means) or other basic estimates (e.g. regression coefficient) AND variation (e.g. standard deviation) or associated estimates of uncertainty (e.g. confidence intervals)
☐	☒	For null hypothesis testing, the test statistic (e.g. *F*, *t*, *r*) with confidence intervals, effect sizes, degrees of freedom and *P* value noted<br>*Give P values as exact values whenever suitable.*
☒	☐	For Bayesian analysis, information on the choice of priors and Markov chain Monte Carlo settings
☒	☐	For hierarchical and complex designs, identification of the appropriate level for tests and full reporting of outcomes
☐	☒	Estimates of effect sizes (e.g. Cohen's *d*, Pearson's *r*), indicating how they were calculated

Our web collection on statistics for biologists contains articles on many of the points above.

Software and code

Policy information about availability of computer code

Data collection	No software was used for data collection.
Data analysis	The source code and tutorials of scSemiProfiler (software reported in this manuscript) are publicly available at https://github.com/mcgilldinglab/scSemiProfiler. scSemiprofiler is implemented using Python 3.9, installed via Anaconda (https://www.anaconda.com/), and Pytorch 1.12.1 (https://pytorch.org/).<br><br>Other tools used for downstream analysis include:<br>scikit-learn (v1.3.2) (https://scikit-learn.org/stable/)<br>SCANPY (v1.9.6) (https://scanpy.readthedocs.io/en/stable/)<br>GSEApy (v1.0.4) (https://gseapy.readthedocs.io/en/latest/introduction.html)<br>Faiss (v1.7.4) (https://github.com/kyamagu/faiss-wheels)<br>R (v4.3.0) (https://www.r-project.org/)<br>CellChat (v1.6.1) (http://www.cellchat.org/)<br>Monocle 3 (v1.3.1) (https://cole-trapnell-lab.github.io/monocle3/)<br><br>For benchmarking our deconvolution performance, we used the following methods:<br>CIBERSORTx (https://cibersortx.stanford.edu/)<br>Bisque (https://github.com/cozygene/bisque)<br>DWLS (https://github.com/dtsoucas/DWLS)<br>TAPE (https://github.com/poseidonchan/TAPE)

Scaden (https://github.com/poseidonchan/TAPE and https://github.com/KevinMenden/scaden).
MuSiC & NNLS (https://github.com/xuranw/MuSiC)
EPIC (https://github.com/GfellerLab/EPIC)

For manuscripts utilizing custom algorithms or software that are central to the research but not yet described in published literature, software must be made available to editors and reviewers. We strongly encourage code deposition in a community repository (e.g. GitHub). See the Nature Portfolio guidelines for submitting code & software for further information.

Data

Policy information about availability of data

All manuscripts must include a data availability statement. This statement should provide the following information, where applicable:

- Accession codes, unique identifiers, or web links for publicly available datasets
- A description of any restrictions on data availability
- For clinical datasets or third party data, please ensure that the statement adheres to our policy

All three datasets associated with this study are publicly available.

The preprocessed COVID-19 dataset can be downloaded from Array Express under accession number EMTAB-10026 (https://www.ebi.ac.uk/biostudies/arrayexpress/studies/E-MTAB-10026).

The count expression matrices of the colorectal cancer dataset are available through Synapse under the accession codes syn26844071 (https://www.synapse.org/#!Synapse:syn26844071/wiki/615389).

The raw count iMGL bulk and single-cell data of the iMGL dataset can be downloaded from the Gene Expression Omnibus (GEO) repository under accession number GSE226081 (https://www.ncbi.nlm.nih.gov/geo/query/acc.cgi?acc=GSE226081).

The hamster bulk and single-cell data can be downloaded from the GEO repository under accession GSE200596 (https://www.ncbi.nlm.nih.gov/geo/query/acc.cgi?acc=GSE200596)

Research involving human participants, their data, or biological material

Policy information about studies with human participants or human data. See also policy information about sex, gender (identity/presentation), and sexual orientation and race, ethnicity and racism.

Reporting on sex and gender	N/A
Reporting on race, ethnicity, or other socially relevant groupings	N/A
Population characteristics	N/A
Recruitment	N/A
Ethics oversight	N/A

Note that full information on the approval of the study protocol must also be provided in the manuscript.

Field-specific reporting

Please select the one below that is the best fit for your research. If you are not sure, read the appropriate sections before making your selection.

[x] Life sciences [ ] Behavioural & social sciences [ ] Ecological, evolutionary & environmental sciences

For a reference copy of the document with all sections, see nature.com/documents/nr-reporting-summary-flat.pdf

Life sciences study design

All studies must disclose on these points even when the disclosure is negative.

Sample size	The study involves three publicly available datasets as mentioned in the 'Data availability' section of our manuscript. The COVID-19 dataset comprises 143 samples with 647,366 cells. The colorectal cancer dataset includes 189 samples containing 373,058 cells. The iMGL dataset has 25 single-cell samples with corresponding bulk data. We used the 74,372 cells labeled by the dataset provider. The hamster dataset has 16 paired single-cell and bulk samples. The single-cell dataset has 18,423 cells.
Data exclusions	We processed all datasets with our standard preprocessing pipeline detailed in the Methods section of our paper. For the colorectal cancer dataset, low-quality cells were removed based on the following criteria: expressing fewer than 200 genes, having fewer than 2,000 read counts, containing more than 20% mitochondrial reads, or expressing more than 6,000 genes. The cell filtering for the other two datasets was

	performed by the dataset providers. For all three datasets, we removed low quality samples with fewer than 1000 cells. After filtering, the COVID-19 dataset has 124 samples and 637,144 cells. The colorectal cancer dataset has 112 samples and 214,358 cells. The iMGL dataset still has 25 samples and 74,372 cells.
Replication	All computational experiments can be replicated using the code provided in our GitHub repository: https://github.com/mcgilldinglab/scSemiProfiler
Randomization	When evaluating the representative sample selection task, we used a random selection strategy to represent a theoretical upper bound.
Blinding	Blinding is not applicable to our method development work.

Reporting for specific materials, systems and methods

We require information from authors about some types of materials, experimental systems and methods used in many studies. Here, indicate whether each material, system or method listed is relevant to your study. If you are not sure if a list item applies to your research, read the appropriate section before selecting a response.

Materials & experimental systems

n/a	Involved in the study	
☒	☐	Antibodies
☒	☐	Eukaryotic cell lines
☒	☐	Palaeontology and archaeology
☒	☐	Animals and other organisms
☒	☐	Clinical data
☒	☐	Dual use research of concern
☒	☐	Plants

Methods

n/a	Involved in the study	
☒	☐	ChIP-seq
☒	☐	Flow cytometry
☒	☐	MRI-based neuroimaging

Plants

Seed stocks	N/A
Novel plant genotypes	N/A
Authentication	N/A
